# Prevalence of *HLA-B27* in the New Zealand population: effect of age and ethnicity

**DOI:** 10.1186/ar4341

**Published:** 2013-10-22

**Authors:** Rebecca L Roberts, Mary C Wallace, Gregory T Jones, Andre M van Rij, Tony R Merriman, Andrew Harrison, Douglas White, Lisa K Stamp, Daniel Ching, John Highton, Simon M Stebbings

**Affiliations:** 1 Department of Surgical Sciences, Dunedin School of Medicine, University of Otago, PO Box 913, Dunedin 9054, New Zealand; 2 Department of Biochemistry, University of Otago, PO Box 56, Dunedin 9056, New Zealand; 3 Department of Medicine, University of Otago, PO Box 7343, Wellington 6242, New Zealand; 4 Wellington Regional Rheumatology Unit, Hutt Hospital, Private Bag 31907, Lower Hutt 5040, New Zealand; 5 Department of Rheumatology Waikato Hospital, Private Bag 3200, Hamilton 3240, New Zealand; 6 Department of Rheumatology and Immunology Christchurch Hospital, PO Box 4710, Christchurch 8140, New Zealand; 7 Department of Medicine, Christchurch School of Medicine, PO Box 4345, Christchurch 8140, New Zealand; 8 Department of Rheumatology Timaru Hospital, Private Bag 911, Timaru, Dunedin, New Zealand; 9 Department of Rheumatology, Dunedin Hospital, Private Bag 1921, Dunedin 9054, New Zealand; 10 Department of Medicine, Dunedin School of Medicine, PO Box 56, Dunedin 9054, New Zealand

## Abstract

**Introduction:**

*HLA-B27* genotyping is commonly used to support a diagnosis of ankylosing spondylitis (AS). A recent study has suggested that *HLA-B27* may adversely affect longevity. The objectives of this study were to determine, for the first time, the prevalence of *HLA-B27* in the New Zealand population, and to test whether *HLA-B27* prevalence declines with age.

**Methods:**

117 Caucasian controls, 111 New Zealand Māori controls, and 176 AS patients were directly genotyped for *HLA-B27* using PCR-SSP. These participants and a further 1103 Caucasian controls were genotyped for the *HLA-B27* tagging single nucleotide polymorphisms (SNPs) rs4349859 and rs116488202. All AS patients testing positive for *HLA-B27* of New Zealand Māori ancestry underwent high resolution typing to determine sub-allele status.

**Results:**

*HLA-B27* prevalence was 9.2% in New Zealand Caucasian controls and 6.5% in Māori controls. No decline in *HLA-B27* prevalence with age was detected in Caucasian controls (p = 0.92). Concordance between *HLA-B27* and SNP genotypes was 98.7-99.3% in Caucasians and 76.9-86% in Māori. Of the 14 AS patients of Māori ancestry, 1 was negative for *HLA-B27*, 10 were positive for HLAB*2705, and 3 positive for HLAB*2704. All cases of genotype discordance were explained by the presence of HLAB*2704.

**Conclusions:**

*HLA-B27* prevalence in New Zealand Caucasians is consistent with that of Northern European populations and did not decline with increasing age. In Māori with AS who were *HLA-B27* positive, 76.9% were positive for HLA-B*2705, suggesting that genetic susceptibility to AS in Māori is primarily due to admixture with Caucasians.

## Introduction

Human leukocyte antigen (HLA) class I molecule *HLA-B27* was the first genetic risk factor identified as associating with ankylosing spondylitis (AS) and remains the most important risk locus for this archetypal spondyloarthropathy [1]. Twin and family studies estimate that *HLA-B27* accounts for 20 to 50% of the total genetic risk of AS [2] and confers an odds ratio in European Caucasians >100 for AS [1]. To date, 100 suballeles of *HLA-B27* have been described [1]. Of these suballeles, HLA-B*2701, HLA-B*2702, HLA-B*2704, HLA-B*2705, and HLA-B*2707 have been associated with AS [3]. As yet there are few data on whether the other suballeles are associated with altered disease susceptibility.

Data from murine models suggest that HLA-B27 is directly involved in the pathogenesis of AS and it is recognised in human populations that the prevalence of the gene reflects the prevalence of AS [1]. However, the mechanism by which this HLA protein contributes to disease remains a source of intense speculation. Hypotheses for the role of HLA-B27 in the pathogenesis of AS can be broadly divided into those related to aberrant processing of antigenic peptides and endoplasmic reticulum stress resulting from a tendency for HLA-B27 to misfold and form homo-dimers. The molecular mimicry/cross-tolerance theory relating to specific bacterial antigens is currently less favoured [1].

The association of *HLAB-27* within the broader group of spondyloarthropathies (SpA) varies significantly, ranging from <50% in psoriatic, enteropathic, and inflammatory bowel disease-associated SpA, to 80% in reactive arthritis, to >95% in AS. The frequency of the *HLA-B27* allele also varies widely across populations [4]. Both a north–south gradient and an east–west gradient have been observed for *HLA-B27* prevalence in the Northern Hemisphere. It is hypothesised that these gradients result from the negative selection pressure exerted by malaria [5]. In regions where malaria is endemic, the prevalence of *HLAB-27* is low, and *vice versa*[5]. *HLA-B27* is highly prevalent in Northern Eurasia and North America, with 10 to 16% of Norwegians, Swedes and Icelanders and 25 to 50% of Inuit, Yupik and Indigenous Northern Americans (for example, Haida and Bella Coola) carrying this allele [4]. *HLA-B27* prevalence decreases to 9.5% in the United Kingdom [6], and further decreases to 2 to 6% in Mediterranean regions [4]. In a similar manner, *HLA-B27* prevalence decreases from west to east. In Southeast Asia prevalence of *HLA-B27* can exceed 12%, but in mainland China the range is between 2 and 6% [4]. *HLA-B27* prevalence also varies significantly within the Pacific Islands. In Melanesia the prevalence is high, whereas *HLA-B27* is uncommon in Micronesia and absent in unmixed native populations of Southeast Polynesia [4].

At present there is a paucity of prevalence data regarding the *HLA-B27* in the New Zealand population, including indigenous Maori. Three previous studies have included New Zealanders. In the first study, Gonzalez-Roces and colleagues conducted a worldwide survey of *HLA-B27* polymorphisms, and included 12 disease-free New Zealand Māori who tested positive for *HLA-B27*[3]*.* The second study related to the prevalence of HLA-B27 in patients presenting to an acute eye service with a history of bilateral or recurrent anterior uveitis. In this study 124 consecutive patients undergoing uveitis screening were typed for HLA-B27. Of these patients, 44 were positive for HLA-B27 and 41% (*n* = 18 out of 44) showed early radiologic evidence of AS [7]. No ethnicity data were available for this patient dataset. In the third study, 116 patients with SpA, 23 patients with uveitis, and 47 patients of unknown disease status were identified by the New Zealand Blood Service as serologically positive for HLA-B27 and sequenced to determine the suballele prevalence [8]. No healthy controls were included in either of the two latter studies, and none of the SpA patients in the study of Stewart were assessed clinically to determine whether they met classification criteria for AS [8]. None of these three studies provided prevalence data for *HLA-B27* in New Zealand. Given that *HLA-B27* remains the most important genetic risk factor for the development of AS, and testing is frequently used to assist diagnosis, there is clinical relevance to establishing the prevalence of this *HLA* allele, which could help with the planning of health resource allocation in New Zealand.

In countries with a high prevalence of AS, dedicated clinics are increasingly being established to assist with the assessment of patients an initiation of anti-tumour necrosis factor therapies. Furthermore, a recent *HLA-B27* prevalence study from the United States reported a significant decline in the prevalence of *HLA-B27* with age [9]. The overall age-adjusted *HLA-B27* prevalence in the United States was estimated at 6.1% (95% confidence interval = 5.3 to 10.4), but when age groups were examined separately the prevalence in 40 to 49 year olds was 8.1% (95% confidence interval = 5.8 to 11.2) and dropped to 3.6% (95% confidence interval = 2.2 to 5.8) in 50 to 69 year olds [9]. This observation raises the possibility that *HLA-B27* status has a detrimental effect on longevity.

Our study had three aims. The first aim was to determine the prevalence of *HLA-B27* in three large control datasets (two Caucasian, one New Zealand Māori) and one AS dataset using single-specific primer polymerase chain reaction (PCR-SSP) and the *HLA-B27* tagging single nucleotide polymorphisms (SNPs) rs4349859 and rs116488202. The second aim was to determine *HLA-B27* suballeles in AS patients of New Zealand Māori ancestry. The third aim was to investigate whether the decline in *HLA-B27* prevalence with increasing age, observed in US data, is also observed in the New Zealand population.

## Methods

### Study participants

A total of 176 patients with AS, fulfilling the modified New York criteria [10], were genotyped as part of an ongoing New-Zealand-wide longitudinal study of AS. All patients with AS were assessed clinically at the time blood samples were obtained for genetic testing. The assessment provided an indication of disease severity at the time of enrolment into the genetic study. Disease activity was assessed using the Bath Ankylosing Spondylitis Disease Activity Index (range 0 to 10) [11], and C-reactive protein (mg/l) was measured at Southern Community Laboratories (normal range for laboratory <1 mg/l). The Bath Ankylosing Spondylitis Functional Index (range 0 to 10) [12] assessed functional limitation in the AS patients. Following a clinical examination, spinal mobility was assessed using the Bath AS Metrology Index (range 0 to 10), higher scores indicating increasing limitation of mobility [13]. Treatment with anti-tumour necrosis factor alpha inhibitors was recorded in all patients. This acts a marker of disease severity, since all patients receiving such treatment in New Zealand are required to have bilateral sacroiliitis on X-ray, a Bath Ankylosing Spondylitis Disease Activity Index >6.0 and limitation of spinal flexion and/or chest expansion.

Two Caucasian healthy control datasets (A1, 485 controls; and A2, 735 controls) and 111 New Zealand Māori controls were included in this study. The A1 and A2 datasets were screened for relevant diseases using self-reporting questionnaires and for A2 controls, physiological (ankle brachial pressure index), anthropomorphic (height, weight and waist–hip ratio) and ultrasound (aorta and carotid arteries) assessments of vascular disease status along with a review of their current medications were also performed. Each control dataset has been described in detail elsewhere [14,15]. A summary of the basic demographics of patients and controls is presented in Table 1. Self-reported ancestry data were available for all study participants. To be included as a Māori control or Māori AS patient, an individual needed a minimum of two Māori grandparents. Informed written consent was obtained from all study participants, and ethical approval for this study was granted by the Upper South and Lower South Regional Ethics Committees of New Zealand.

**Table 1:** Basic demographics of the New Zealand control and ankylosing spondylitis datasets

**Demographic**	**Caucasian controls, A1**	**Caucasian controls, A2**	**New Zealand Māori controls**	**AS patients (New York criteria)**
	**(*n*** **= 485)**	**(*n*** **= 735)**	**(*n*** **= 111)**	**(*n*** **= 176)**
Males (%)	200/485 (41.2)	570/735 (77.5)	30/111 (27.0)	113/152 (74.3)
Mean age (years)	44.9	69.6	41.7	46.7
Ethnicity	100% Caucasian	100% Caucasian	100% New Zealand Māori	92.1% Caucasian, 7.9% Māori
Mean (SD) BASDAI	–	–	–	4.8 (2.5)
Mean (SD) BASFI	–	–	–	4.4 (2.8)
Mean (SD) BASMI	–	–	–	3.2 (2.6)
Mean (SD) CRP	–	–	–	11.5 (13.7)
Patients receiving TNFi (%)	–	–	–	69 (39.2)
Comorbidities (%)				
Osteoarthritis	3 (0.6)	125 (29.0)	–	–
Rheumatoid arthritis	1 (0.2)	28 (6.5)	9 (8.1)	–
Other forms of arthritis	–	10 (2.3)	–	–
IBD	1 (0.2)	–	–	–

BASDAI, Bath Ankylosing Spondylitis Activity Index; BASFI, Bath Ankylosing Spondylitis Functional Index; BASMI, Bath Ankylosing Spondylitis Metrology Index; CRP, C-reactive protein; IBD, inflammatory bowel disease; SD, standard deviation; TNFi, tumour necrosis factor inhibitors.

### DNA extraction and SNP genotyping

DNA was obtained from peripheral blood using guanidine isothiocyanate extraction. The major histocompatibility complex (MHC) SNP rs4349859 has been found to tag the major Caucasian AS-associated suballeles HLA-B*2702, HLA-B*2705 and HLA-B*2708 with 98% sensitivity and 99% specificity. However, this SNP does not tag the African AS-associated suballele HLA-B*2703, nor the Asian suballeles HLA-B*2704, HLA-B*2706, and HLA-B*2707[16]. More recently another tagging SNP, rs116488202, has been identified that may accurately tag both Caucasian and Asian *HLA-B27* suballeles [17]. Genotyping of rs4349859 and rs116488202 was carried out using a predesigned TaqMan® SNP genotyping assay (Assay ID number C__28023949_10) and a custom designed TaqMan® SNP genotyping assay (Assay ID number AHCS8KA), respectively, as per the manufacturer’s instructions (Applied Biosystems, Carlsbad, California, USA). Briefly, the assays were performed in a total reaction volume of 5 μl containing 2.5 μl of 2× TaqMan® Universal Master Mix (Roche Molecular Systems Inc, Branchburg, NJ, USA), 0.25 μl of 20× working mix of SNP genotyping assay and 12 ng genomic DNA. The PCR was performed in a Roche LightCycler 480 real-time PCR system, with an activation step of 10 minutes at 95°C, following by 40 cycles of denaturation (15 seconds at 92°C) and annealing/extension (1 minute at 60°C). Genotypes were assigned using the endpoint genotyping analysis software. The accuracy of the TaqMan® SNP assays was confirmed by repeat analysis of 10% of samples. Concordance between original and repeat genotype calls was 100% for both assays. PLINK software [18] was used to test for deviations in Hardy–Weinberg equilibrium.

Our control datasets (A1, 485 controls vs. A2, 735 controls) had 99% power to detect the effect size of odds ratio = 0.4 (α = 0.05) for age, previously reported by Reveille and colleagues [9].

#### HLA-B27 *typing*

Direct typing of the *HLA-B27* allele was performed by the Canterbury Health Laboratories (Christchurch, New Zealand) and Waikato Hospital Laboratory (Hamilton, New Zealand) on 117 Caucasian controls, 111 New Zealand Māori controls, and 176 AS patients. All AS patients of New Zealand Māori ancestry who tested positive for *HLA-B27* by PCR-SSP underwent high-resolution *HLA* typing to determine their *HLA-B27* suballele status. *HLA-B27* suballeles were determined by the Immunogenetics and Transplantation Laboratory (University of California, San Francisco, CA, USA) using DNA sequence-based typing.

## Results

A total of 117 Caucasian controls, 111 New Zealand Māori controls and 176 AS patients were typed for *HLA-B27* using PCR-SSP. The prevalence of *HLA-B27* in New Zealand Caucasians, Māori, and AS patients was noted as 7.7%, 6.5% and 93.2%, respectively. These study participants, as well as 1,103 additional Caucasian controls, were then genotyped for the *HLA-B27* tagging SNPs rs4349859 and rs116488202 (Table 2). The concordance between *HLA-B27* and tagging SNP genotypes was 100% in New Zealand Caucasian controls and 98.7 to 99.3% in New Zealand Caucasians with AS, but declined to 76.9 to 85.7% in AS patients and controls of New Zealand Māori ancestry (Table 2). The Caucasian controls comprised two datasets (A1 and A2). The combined prevalence of *HLA-B27* in these datasets, inferred by rs4349859 genotype, was 9.2% (11/1,220). Significant deviation from Hardy–Weinberg equilibrium was observed for rs4349859 in the AS dataset (*P* = 2.09 × 10^–11^), whereas no deviations from Hardy–Weinberg equilibrium were observed in any of the control datasets for this SNP (*P* >0.05).

**Table 2:** **Concordance between *HLA-B27 *** **and tagging SNP rs4349859 and rs116488202 genotypes in New Zealand controls and ankylosing spondylitis patients**

**Phenotype**		**Frequency (*n *** **(%))**			**Genotype concordance (%)**	
		***HLA-B27***-**positive**^**α**^	**rs4349859-positive**^**b**^	**rs116488202-positive**	**rs4349859**	**rs116488202**
Controls	Caucasian	9/117 (7.7)	9/117 (7.7)	9/117 (7.7)	100.0	100.0
	New Zealand Māori	7/107 (6.5)	6/107 (5.6)	6/107 (5.6)	85.7	85.7
AS	Overall	164/176 (93.2)	159/176 (90.3)	159/176 (90.3)	96.9	97.6
	Caucasian	151/162 (93.2)	149/162 (91.9)^c^	150/162 (92.6)^d^	98.7	99.3
	New Zealand Māori	13/14 (92.9)	10/14 (71.4)	10/14 (71.4)	76.9	76.9

AS, ankylosing spondylitis; SNP, single nucleotide polymorphism.

^α^HLA-B27 status determined by human leucocyte antigen (HLA) typing.

^b^Study participants were positive if heterozygous or homozygous for the minor allele of rs4349859 or rs116488202.

^c^One patient was HLA-B*2705-positive by typing but was negative for the minor allele of both tagging SNPs.

^d^One patient was HLA-B*2705-positive by typing but was negative for the minor allele of rs4349859.

A total of 14 patients in the AS dataset were identified as New Zealand Māori by self-report (Table 1). Of these 14 patients one was *HLA-B27*-negative, and 13 were *HLA-B27*-positive. All 14 patients went forward to DNA sequence-based typing to determine their *HLA-B27* suballele status. Ten were positive for the European suballele HLAB*2705, and three for the Asian suballele HLAB*2704.

The two Caucasian control datasets, A1 and A2, were used to determine whether *HLA-B27* prevalence declined with increasing age. Participants in the A1 dataset had a mean age of 44.9 years whereas participants in the A2 dataset had a mean age of 69.9 years (Table 3). *HLA-B27* prevalence for each dataset was inferred from the rs4349859 genotype. In the A1 dataset, 9.1% (44/485) were heterozygous or homozygous for the minor (A) allele compared with 9.2% (68/735) in the A2 dataset (*P* = 0.92) (Table 3).

**Table 3:** **Genotype and minor allele frequency of the *HLA-B27 *** **tagging SNP rs4349859 in New Zealand Caucasian controls stratified according to age**

**Dataset**	**Mean age (years)**	**rs4349859 genotype frequency**			**MAF**^**a**^	**Inferred *HLA-B27 *** **prevalence**^**b**^	***P *** **value**
		**GG**	**AG**	**AA**			
A1 (*n* = 485)	44.9	441 (0.909)	42 (0.087)	2 (0.004)	46 (0.047)	44 (0.091)	0.92
A2 (*n* = 735)	69.9	667 (0.907)	65 (0.088)	3 (0.004)	71 (0.048)	68 (0.092)	

SNP, single nucleotide polymorphism.

^a^Minor allele frequency.

^b^Determined by adding together the rs4349859 AG and AA genotypes.

## Discussion

To our knowledge this is the first study to investigate *HLA-B27* in a large number of Caucasian (*n* = 1,220) and Māori (*n* = 111) controls to determine prevalence of this AS-associated allele in the New Zealand population. The prevalence of *HLA-B27* in New Zealand Caucasian controls (9.2%) was similar to the prevalence of 9.5% previously reported in a dataset of 5,926 UK controls [6]. Similarly, the overall *HLA-B27* prevalence of 93.2% in our predominately Caucasian AS patient dataset was consistent with the prevalence previously reported in overseas Caucasian AS datasets [6]. There is a paucity of existing data regarding the prevalence of AS in Māori.

Concordance between the tagging SNPs and *HLA-B27* genotypes was 98.7% for rs4349859 and 99.3% for rs116488202 in the Caucasian AS patients but declined to 76.9% for both SNPs in AS patients of New Zealand Māori ancestry (Table 2). A previous study found that SNP rs116488202 tagged *HLA-B27* more accurately than rs4349859 in both Caucasians and Asians [17]. In our study, whilst SNP rs116488202 tagged HLA-B27 more accurately for Caucasians this was not the case for New Zealand Māori. Subsequent high-resolution *HLA* typing found the three Māori patients with discordant genotypes were all positive for the Asian AS-associated HLAB*2704 suballele, which neither rs4349859 nor rs116488202 genotyping identified [16]. To assess the ability of rs116488202 to tag *HLA-B27* suballeles more fully, we sent DNA from three Asian patients (one Filipino, one Han Chinese, one Thai) with AS for high-resolution HLA typing. One patient was found to carry HLAB*2705 and two patients tested positive for HLAB*2704. Subsequent rs116488202 genotyping was only able to identify the patient who carried the suballele HLAB*2705 as HLA-B27-positive. These findings suggest that whilst rs116488202 is a more accurate tagging SNP for HLA-B27 in Caucasians, it does not tag HLA-B27 any more strongly than rs43439859 in Asians or New Zealand Māori. However, it is important to note that our study only had 14 New Zealand Māori and three Asian participants, and therefore does not have the power to allow us to draw any firm conclusions on the relative performance of these two tagging SNPs in individuals of Māori or Asian ancestry.

Direct *HLA* typing by PCR-SSP typically costs around NZD$55 per sample, requires 2 μg DNA, and has a 14-day turnaround. In comparison, genotyping by TaqMan® costs NZD$5 per sample, requires 8 to 16 ng DNA, and can be completed within 4 to 8 hours. Given the high concordance between *HLA-B27* and SNP genotypes observed in New Zealand Caucasians (Table 2), genotyping for rs4349859 or rs116488202 could serve as a rapid and inexpensive way of reliably establishing *HLA-B27* status in predominately Caucasian AS datasets.

The distribution of AS-associated *HLA-B27* suballeles differs significantly across populations. HLAB*2705 is found in all racial groups and thus is widely considered to be the ancestral suballele from which all other variants of *HLA-B27* have arisen. HLAB*2705 is the most common suballele in Caucasians, but a second, Caucasian-specific suballele (HLAB*2702) is also seen, albeit at a much lower frequency. HLAB*2704 is the most common AS-associated suballele in East Asians but has not been reported in Caucasians [3]. Of the 11 Māori AS patients who underwent high-resolution typing, 10 carried HLAB*2705 and three carried HLAB*2704. In the only previous study of *HLAB-27* prevalence to include New Zealand Māori [3], seven of 12 (58%) individuals carried the HLAB*2705 allele and five individuals (42%) carried the HLAB*2704 allele. The presence of the Asian-specific suballele HLAB*2704 in New Zealand Māori supports a recent investigation into genetic structure of Pacific Islanders, suggesting Polynesians are closely related to Taiwanese Aboriginal populations [19], a finding strongly supported by anthropological evidence and a common Austronesian language structure. A study of Taiwanese Aboriginal populations demonstrated a differential prevalence of *HLA-B27* amongst different tribes, with a high prevalence of *HLA-B27* (9.2%) in Atayal Aborigines, 2.1% in the Paiwan and none in the Rukai. HLAB*2704 was the only suballele identified in the aboriginal population [20]. In the current study, AS patients of New Zealand Māori ancestry have a low prevalence of this HLAB*2704, indicating that genetic susceptibility to AS in this population is primarily due to significant recent admixture with Caucasians.

The final aim of our study was to investigate the potential link between *HLA-B27* status and longevity. A recent study of *HLA-B27* in 2,320 US adults reported a significant decline in the prevalence of the *HLA-B27* allele with age, which remained significant after adjustment for sex and race [9]. Although the decreasing linear trend in *HLA-B27* prevalence with age was not entirely consistent in this dataset [9], the result suggests that *HLA-B27* may negatively impact on longevity, a potentially devastating observation, given that *HLA-B27* is one of the most widely employed clinical genetic tests. Previous investigations offer a possible biological rationale for this observation, although clinical studies following patients with AS have actually shown a reduced standardised mortality ratio in patients with AS [21]. *HLA-B27* has been shown to increase susceptibility to intracellular bacterial pathogens that could in turn increase the risk of atherosclerosis and valvular heart disease, and thus reduce life expectancy. In the current study, however, we found no evidence of a similar decline in *HLA-B27* prevalence in two New Zealand Caucasian datasets of significantly different mean age.

## Conclusions

*HLA-B27* is commonly tested to assist in the diagnosis of AS. Despite the clinical relevance of this locus there is a paucity of prevalence data for *HLA-B27* in the New Zealand population. Our study has established, for the first time, the prevalence of *HLA-B27* in New Zealand Caucasians and Māori. We have found that the prevalence of *HLA-B27* in New Zealand Caucasians is comparable with the prevalence rates observed in Northern European populations, and did not decline with increasing age, suggesting that *HLA-B27* does not adversely affect longevity in the New Zealand Caucasians. Furthermore, the prevalence of *HLA-B27* allele in New Zealand Māori controls (6.5%) was higher than expected. This increase in *HLA-B27* prevalence and the preponderance of the AS-associated suballele in HLAB*2705 in Māori patients indicates that AS susceptibility in New Zealand Māori is largely due to admixture with Caucasians. Finally the high prevalence of *HLA-B27* in the studied population implies a similarly high prevalence of AS in New Zealand. Since AS is associated with both significant negative health and socioeconomic effects, this finding will prove helpful in planning the allocation of publicly funded health resources in New Zealand.

## Abbreviations

AS: Ankylosing spondylitis; HLA: Human leucocyte antigen; PCR: Polymerase chain reaction; SNP: Single nucleotide polymorphism; SpA: Spondyloarthropathies; SSP: Single-specific primer.

## Competing interests

The authors declare that they have no competing interests.

## Authors’ contributions

RLR and SMS contributed equally to this study. RLR participated in the design of the study, data analysis, and drafting the manuscript. MCW performed the tagging SNP genotyping of patients and controls, and participated in data analysis and the drafting of the manuscript. GTJ and AMvR provided the A2 control cohort. TRM provided the A1 control cohort. AH, DW, LKS, DC, and JH provided AS patients and associated phenotype data. SMS participated in the design of the study, data analysis, drafting of the manuscript, and coordinated patient recruitment from different centres within New Zealand. All authors read and approved the final manuscript.
